# An Evidence-Based Exercise Regimen for Patients with Mild to Moderate Parkinson’s Disease

**DOI:** 10.3390/brainsci3010087

**Published:** 2013-01-16

**Authors:** Sanjay Salgado, Nori Williams, Rima Kotian, Miran Salgado

**Affiliations:** 1 Weill Cornell Medical College of Cornell University, New York, NY 10065, USA; E-Mail: smsalgado@gmail.com; 2 Department of Neurosciences, New York Methodist Hospital, Brooklyn, NY 11215, USA; E-Mail: williams.nori2@gmail.com; 3 Metro SportsMed, Brooklyn, NY 11215, USA; E-Mail: rima.kotian@gmail.com

**Keywords:** Parkinson’s disease, exercise, rehabilitation

## Abstract

Parkinson’s disease (PD) is a neurological disorder that is manifested in the form of both motor and non-motor symptoms such as resting tremor, bradykinesia, muscular rigidity, depression, and cognitive impairment. PD is progressive in nature, ultimately leading to debilitating disruption of activities of daily living. Recently, a myriad of research has been focused on non-pharmacological interventions to alleviate the motor and non-motor symptoms of the disease. However, while there is a growing body of evidence supporting exercise as a viable therapy option for the treatment of Parkinson’s disease, there is a lack of literature enumerating a specific exercise sequence for patients with PD. In this literature review, we analyze the success of specific modalities of exercise in order to suggest an optimal exercise regimen for Parkinson’s disease patients.

## 1. Introduction

Parkinson’s Disease (PD) is a debilitating illness affecting between 4.1 and 4.6 million individuals over the age of 50 [1], making it the second most common neurodegenerative disorder after Alzheimer’s disease. The initial clinical signs appear after the degeneration of about 60% of the dopaminergic neurons of the substantia nigra [2], resulting in motor dysfunction that manifests with a tetrad of primary symptoms—resting tremors, bradykinesia, muscular rigidity and postural instability. Research has suggested that non-motor symptoms, such as constipation, olfactory dysfunction and depression, also occur, and may precede motor dysfunction by several years [3]. Progressive in nature, PD tends to worsen with time, leading to a general decrease in activity [4], an increased risk of falling, immobility, and cognitive impairment [5].

Many aspects of patient’s lives are affected by this progressive disease; over three-quarters of patients complain of difficultly walking, rising from chairs, clumsy movements, a lack of energy and the need to expend extra physical effort to accomplish daily tasks [6]. As such, there is a considerable benefit to identifying supplemental therapies that, in conjunction with pharmacologic and surgical treatments, may increase patient strength and endurance.

### History of the Role of Exercise in Parkinson’s

The benefits of exercise in PD have not always been well characterized. Despite the American Academy of Neurology encouraging the use of exercise as an adjunctive therapy for Parkinson’s patients in the 1990s [7], systemic reviews from the Cochrane collaboration released in 2001 found insufficient evidence to support or refute the efficacy of physiotherapy in PD [8,9]. Although most of the individual trials analyzed in these reviews appeared to find a beneficial effect of physiotherapy, it was determined that many of the studies had methodological flaws and biases that prevented any firm conclusions of the validity of physiotherapy [8]. Interestingly, this led to a situation in which physicians were instructed to encourage regular exercise, despite little evidence of its efficacy in slowing disease progression or improving activities of daily living [10]. 

However, more recent studies suggest that there are indeed benefits of physical activity in PD patients. A recent meta-analysis supported exercise as being beneficial with regards to physical functioning, health-related quality of life, strength, balance, and gait speed for PD patients [11]. A large study following 438 PD patients found after four years, that mortality was lower for regularly exercising patients, even for those who could not walk independently [12]. Despite affirming the benefits of exercise, there is little consensus of dosages and types of exercise needed to target the wide range of symptoms that present with PD. This review attempts to highlight the potential benefits of cardiovascular, balance, and resistance training while extracting the most efficacious exercise strategies to mitigate the bradykinesia, muscle weakness, and balance impairment experienced by PD patients.

Furthermore, to apply these findings in a clinical setting to improve the quality of life for Parkinson’s disease patients (taking into account the importance of empowering patients with PD to self-manage their disease to some extent [13]), we have used the existing literature to develop a high-yield exercise regimen designed to maximize balance, coordination and functional strength for patients with mild to moderate disease. We focus on exercises that can readily be translated into a home-based program, noting that a Cochrane review found older adults are more likely to adhere to home-based programs than center-based programs [14]. Acknowledging that there is no “one size fits all” exercise prescription for such a variable and progressive disease, we advocate this regimen not as a universally applicable exercise plan, but rather as a template to be modified by physicians and physiotherapists to best suit the needs of patients.

## 2. Exercise in Parkinson’s Disease

### 2.1. Exercise and Risk of Disease

Exercise appears to have a neuroprotective effect against developing PD. Prospective cohort studies have found that men [15,16,17] and women [16,17] who partake in moderate [16,17] and strenuous [15,16,17] (but not light [16,17]) exercise (including swimming, tennis, basketball, cycling and running) appear to have a significantly decreased incidence in developing PD, although this is not a universal finding [18]. Interestingly, higher levels of activity during 35–39 years of age—decades before the average age of onset of the disease—significantly reduces risk of acquiring the disease [17]. Evidence from animal models support these suggested neuroprotective effects of exercise against developing PD, highlighting the influence of exercise on neuroplasticity and self-repair [19,20].

While the underlying mechanisms for these potential neuroprotective effects are unclear, elevated plasma uric acid levels, seen in vigorous (but not light) exercise [21], is hypothesized to play a role in the decreased risk and slower progression of the disease (for reviews, see [22,23,24]). It should be noted that these findings could be due to reverse causality—*i.e.*, preclinical PD may lead to a reduction in activity, rather than a reduction in activity leading to an increased risk for the disease. However, this appears unlikely, as animal models suggest decreased physical activity is not a symptom of the disease itself [25] and human studies have found no noticeable decrease in activity before diagnosis [4]. However, while the benefits of exercise for PD patients have become more widely accepted recently, the most effective exercise sequence has yet to be elucidated. 

### 2.2. Cardiovascular Training for Parkinson’s Patients

Cardiovascular training, as defined as a routine that increases heart rate and oxygen demand, appears to have direct benefits for Parkinson’s patients in a dose-dependent fashion [26]. Interestingly, some of these benefits are evident nearly immediately. After a single session of either speed-dependent treadmill training or limited-progressive treadmill training, patients show improved gait parameters compared to controls and those given only conventional gait training [27]. Similarly, a single session of a high intensity cardiovascular exercise intervention, in the form of assisted cycling, showed reductions in tremor and bradykinesia without excessive fatigue in PD patients [28]. Weeks of treadmill training showed enhanced balance, gait, and improved quality of life [29,30], with the benefits persisting for four weeks [31]. One case study has reported that eight weeks of aerobic exercise improved language and cognition in a PD patient who already had high performance scores in cognitive measures at baseline [32]. In a recent randomized clinical trial, 121 patients with mild to moderate PD who underwent 16 weeks of aerobic endurance exercise (utilizing a treadmill, bicycle, or elliptical trainer) improved overall function, balance, and movement efficiency [11]. Importantly, compared with alternative exercise modalities, this study further suggested aerobic endurance was the most beneficial for walking economy. Case studies of patients (Hoehn and Yahr, H & Y, stages 2–2.5) who underwent still longer training (4 months of cardiovascular training regimens supplemented with an additional 12 months of home exercise) have reported sustained increases in UPDRS, functional performance, and economy of movement during the entire course of training [33]. Coupled with the findings that sedentary, but otherwise healthy adults do not typically increase walking economy with endurance training [34,35], these results suggest that Parkinson’s patients do not simply benefit from exercise to the same extent as healthy individuals [36], but may gain advantages from exercise above and beyond those available to the general population. 

While there is a limited amount of randomized controlled studies confirming the long term benefits of cardiovascular training in PD patients, there is a growing body of data suggesting that this is the case. Patients with mild to moderate PD progression (H & Y stage 1–3) who underwent high intensity treadmill training showed changes in brain plasticity, namely a lengthening of the cortical silent period [26], a measure of corticomotor excitability typically shortened in Parkinson’s patients [37]. Patients that perform cardiovascular exercise also have an increased longevity in mortality studies [12], further implicating the existence of long term benefits.

Less conventional therapies, such as body weight-supported treadmill training, have also been attempted in patients with mild to moderate PD (H & Y stages 1–3), and have led to a greater improvement in ambulation speed, number of steps, and short-step gait compared to conventional physical therapy [38,39], with the benefits lasting for about four months [39]. However, the precise differences between treadmill trainings have been difficult to fully assess, as a study attempting to identify the differences between assisted weight bearing, additional weight bearing, and conventional treadmill training in patients (H & Y stages 1–7) found improvements to balance, UPDRS, and gait regardless of the specific type of treadmill training utilized [40].

### 2.3. Balance Training for Parkinson’s Patients

Postural instability and balance impairments are common symptoms of PD [41], contributing to an increased frequency of falls and injuries [42] which in turn increases morbidity and mortality [43]. The large impact of postural instability on patients is a significant concern, especially considering that dopamine replacement medications are often insufficient to control these deficits [44].

There have been a number of studies examining the best types of training to improve balance and mitigate falls. Patients who participate in balance training have shown improvements in gait and ambulation [45]. Results from 10 weeks of balance and strength training indicated improvement in equilibrium by two distinct mechanisms: (1) training altered the ability to control the motor system when vestibular cues had to be the primary source of reliable feedback; (2) training helped subjects to override faulty proprioceptive feedback and utilize reliable visual or vestibular cues. A larger study indicated that patients participating in balance training, compared to general physical exercises, showed improvements as determined by the Berg Balance Scale, Activities-based Balance Scale, postural transfer test, and number of falls [46]. Furthermore, these improvements were maintained one month post-treatment. 

A meta-analysis found moderate evidence that physical activity and exercise will result in improvements in postural instability and balance task performance, although there was limited evidence to support an improvement in quality of life and falls outcomes in patients with mild to moderate PD [47]. Similarly, another meta-analysis found multifaceted training improved balance-related activity performance, but notes that there was no evidence that the number of falls were impacted [48]. The report also suggests that the inclusion of highly challenging balance training may improve the efficacy of intervention on balance-related activity performance. To significantly address falling, balance training may need to be supplemented with resistance training—a small study suggested that the combination of balance and resistance training improved the balance scores of PD patients significantly more than did balance training alone [49]. 

Similar to the limitations of the studies examining the benefits of cardiovascular exercise, the long term effects of balance training is unclear. However, given the progressive nature of PD, it is likely that to maintain efficacy, the balance training would need to be ongoing. Allen *et al.* (2011) [48] alludes to the problematic nature this situation, noting the difficulty of sustainably maintaining a challenging training regimen in a home-based program. However, other studies have suggested a number of readily accessible and relatively inexpensive options by which patients can reduce balance impairments at home. These include the use of commercially available motion controlled video games [50], which could theoretically allow for a home-based, yet monitorable, form of training. 

Another suggested option is Tai Chi [51]. In healthy patients, Tai Chi has been shown to be more effective in preventing falls in elderly patients compared to conventional physical therapy [52]. In patients with mild to moderate PD, Tai Chi training appears to improve physical function [53] and reduce balance impairments in patients [54]. A large randomized clinical trial investigating the impact of a 24 week Tai Chi class, compared to resistance training or low-intensity stretching found that the Tai Chi significantly improved maximum excursion, directional control, gait and strength measures, and is the only large scale trial to demonstrate a significant reduction in falls as a result of exercise [51]. Overall, this suggests that in mild to moderate PD, Tai Chi is more effective than stretching or resistance-training in improving postural stability.

### 2.4. Resistance Training for Parkinson’s Patients

There is a strong body of evidence that suggests PD not only leads to motor dysfunction, but also adversely affects muscle strength (for review, see [55]). Studies have demonstrated that Parkinson’s patients have decreased isokinetic muscle strength affecting a number of muscle groups, most notably the flexors and extensors of the hip, knee, and wrist [56,57,58,59,60,61]. Furthermore, numerous studies have suggested Parkinson’s patients demonstrate altered isometric ability in the hands [31,62,63,64,65,66] and core muscles [67], including longer times to reach peak torque and contraction, lower rates of force development, and irregular force-time curves, although it should be noted that these are not universal findings [57,68]. While the complete mechanism for this weakness is not fully understood, it likely involves decreased activation of motor neurons due to inadequate basal ganglia stimulation of cortical motor centers, as well as incomplete contractions from action tremors caused by the rhythmic and synchronous discharging of motor units during voluntary activity in Parkinson’s patients [69]. Importantly, studies have suggested that muscle weakness is likely inherent to PD and not merely a secondary consequence of aging or inactivity [70]. Coupled with the finding that healthy older adults already expend close to maximal force production capabilities to navigate stairs and rise from chairs [71], the burden of PD on ADLs from muscle weakness cannot be understated. 

To mitigate these strength deficits, resistance training is an accessible modality of exercise for PD patients. A number of small studies have shown improvements in muscle strength [49,72,73,74], muscular endurance [73,75], neuromuscular function [73,75], muscle force production [76], as well as gait speed and initiation [77] as well as chair rise function [73]. All of these improvements impact functional mobility. Interestingly, studies have even suggested that patients with mild-to-moderate PD can obtain increases in strength similar to that of normal adults of the same age in a resistance training program [77].

Resistance training emphasizing both eccentric muscle contractions, which enable high muscle forces with low metabolic requirements [3], and concentric contractions has been shown to improve muscle hypertrophy, strength, and mobility in persons with PD [49]. A clinical trial measuring the impact of high-force eccentric resistance training on muscle volume, muscle force, and functional status showed improvements in persons with PD, with the eccentric trial group showing significantly improved scores for muscle structure, stair descent, and 6 min walk than those in a standard care group [72]. 

There is a limited amount of research determining the volume of such training necessary to yield optimal results, however it is generally agreed that the most advantageous volume of exercise should maximize intensity while minimizing fatigue [28]. Small trials have suggested that in healthy older patients, three sets of strength and endurance training for selected exercises result in greater gains that single sets [78]. More research on the optimal volume is needed for PD patients, as many of the current studies evaluating resistance training in PD employ a single set regimen. Notably, a pilot study measuring the impact of moderate volume (3 sets of 3 exercises), high load weight training on PD patients showed significant strength increases compared to baseline and was well tolerated by participants [79]. This finding suggests a moderate volume of high intensity exercise may be beneficial and manageable for people with PD. However, this notion has been contested. Schenkman *et al.* [80]. compared home-based exercise to more intensive endurance and balance exercise programs. Initially, the two more intensive regimens yielded higher overall functional gains. However, in the long term, all three programs improved overall function. This finding suggests there is a greater importance in maintaining consistently high levels of physical activity than performing a specific regimen of sets and repetitions. 

### 2.5. LSVT BIG™

Lee Silverman Voice Treatment (LSVT) BIG™, which differs from other forms of physical therapy for PD by focusing on movement amplitude as the sole treatment parameter through high-effort training [81], is a form of PD therapy growing in popularity in the PD patient community. Given the programs rapid expansion, a brief discussion of the program is warranted, as physicians will likely encounter questions about the benefits of the program from patients. The goal of LSVT BIG™ is to overcome deficient speed-amplitude regulation leading to underscaling of movement amplitude at any given velocity. Continuous feedback on motor performance and training of movement perception is used to counteract reduced gain in motor activities resulting from disturbed sensorimotor processing [82].

In a noncontrolled study assessing effects of LSVT BIG™ in individuals with PD, after four weeks of training, subjects demonstrated a modest (12%–14%) increase in velocity of walking and reaching movements [83]. In a rater-blinded Berlin study, LSVT BIG™ was found to be more effective than Nordic walking, a type of fitness walking that forces the patient to utilize upper and lower body muscles simultaneously, when delivered as group training [84]. The beneficial outcome was reflected by improvements in assessments including the UPDRS motor score, standard time-up and go task and 10 m walk [84]. Overall, while these results appear promising, these authors express concern about advocating this program for patients until more literature is available.

## 3. Conclusions

As suggested above, specific modalities of exercise such as cardiovascular training, resistance training, and balance training are able to relieve patients of debilitating motor and non-motor symptoms and allow for increased functional ability to complete ADLs. The most beneficial exercise regimen likely combines these three modes of exercise to reduce both motor and non-motor symptoms. 

Utilizing the evidence available enumerated, we have formulated an exercise regimen tailored to treat the specific symptoms of PD: 

(1) Cardiovascular exercise such as high intensity treadmill training or assisted cycling has been shown to reduce bradykinesia as well as improve gait function and ambulation [28,29,30]. Based on these data, and given the lack of evidence suggesting differences between various types of cardiovascular training, we recommend moderate to high intensity cardiovascular exercise in the form of treadmill training or assisted cycling for up to 30 min per session every other day. 

(2) To increase muscle strength, we recommend moderate volume (3 sets each) high load resistance training 2–3 times per week. It has been suggested that this volume and modality may optimally increase muscle strength without causing excessive fatigue [75,79]. While both concentric contractions and eccentric contractions to will increase strength gains, we recommend an emphasis on eccentric movements to minimize metabolic demand. 

(3) Balance training in the form of Tai Chi should be used to improve postural control and walking ability. A large-scale trial demonstrated that patients performing Tai Chi show improvements in maximum excursion and directional control that were not seen in balance training or strength training [51]. Given that patients can complete Tai Chi at home without the use of clinical equipment, coupled with the benefits to balance and postural control that were not observed with traditional balance training alone, we recommend Tai Chi for 1 h at least twice weekly. 

(4) Given the limited number of clinical trials measuring the efficacy of LSVT BIG™, we express caution in recommending LSVT BIG™ as a supplemental therapy at the current time.

(5) For patients who present with a more advanced stage of PD or cannot adhere to an intensive exercise regimen at home, we recommend physical therapy sessions with a licensed Physical Therapist specialized in PD rehabilitation. 

In a clinical setting, patients who undergo physical therapy consisting on strength exercises, balance and gait training in addition to medication therapy show short and long term increases in quality of life, mobility, walking speed, and activities of daily living (ADLs) compared to patients who only receive medication [85]. Physical therapy appears to bestow short term gains for patients which persist for weeks after training, and while cessation of structured therapy sessions lead to a return to baseline strength within months [86], the benefits to walking speed, activities of daily living, and the Unified Parkinson’s Disease Rating Scale continue to persist [85]. Similar goals should be expected for home-based programs. While there has been some success in employing home-exercise regimen consisting of muscle strengthening, balance exercises, and cardiovascular training to lower rates of falling [43], more research is needed to determine whether other home-based programs can mirror this success. 

## 4. Limitations

Notably, many of the studies referenced in this review were of short duration, highly supervised, facility based, and included a limited amount of participants with mild to moderate disease severity, all of which attest to a need to further research. Since PD is a long-term degenerative disorder, studies with longer durations are essential to evaluate how long a patient should be encouraged to continue on an exercise regimen. Highly supervised programs, such as nearly all of the referenced trials, are less likely to give information about effectiveness of the intervention when put into clinical practice [87,88]. Larger randomized controlled studies are needed to confirm many of the findings highlighted in this review, and to determine whether the exercise regimen presented herein is an appropriate guideline for patients with increased disease severity.
